# Video-assisted surgery via periareolar, mitral valve replacement and papillary muscle relocation with neochordae of PTFE

**DOI:** 10.1186/1749-8090-8-S1-P144

**Published:** 2013-09-11

**Authors:** LM Abreu, MLM Alves, J Honório, DC Ferreira, PN Sampaio, D Bastos, A Bosiger, S Godoy, DC Ferreira, O Souza Neto

**Affiliations:** 1 Department of Cardiovascular Surgery, Hospital Federal dos Servidores, Rio de Janeiro, Brazil

## Background

Cardiac surgery is looking for new techniques, which are less traumatic with better cosmetic and functional results. The minimally invasive mitral valve in the first procedure is rapidly becoming standard access in several centers around the word. The objective of this report is to show that this new technique can lead to an improved quality of life after surgery and therefore a quicker return to everyday life.

## Methods

MFS, 42-year-old female, with history of rheumatic process in childhood. Referred to cardiac surgery in New York Heart Association (NYHA) functional class III and indications of surgical treatment of mitral valve. Reached the right hemi thorax through the 4th right intercostals’ space (RICS) in the nipple-areolar complex after ventilation of the left lung. In the same intercostals’ space, anterior to the axillary middle line, was introduced a 7mm optical trocar to 30 °. At 7th RICS in the anterior axillary line (AAL), we implemented a trocar 7mm which was introduced for insufflation of CO2 and introduction of the soft aspirator. cardiopulmonary bypass (CPB) was established via femoral. In order to maintain ventricular geometry were implanted neochordae PTFE Teflon anchored in the region fibrotic papillary muscle and attached to the mitral annulus in the direction of A1 and A2. The biologic prosthesis n° 29 was implanted with points with braided polyester Teflon in a "U".

## Results

The procedure was successfully performed. The CPB time was 110 minutes (min) and aortic clamping of 95 min. The period of mechanical ventilation was 8 hours, bleeding postoperatively of 190ml. In 36 hours the patient was discharged.

## Conclusion

Minimally invasive surgery video assisted with periareolar approach for treatment of mitral valve with papillary muscle repositioning is technique with potential to reduce the recovery time with the advantages of a less invasive procedure for the patient.

